# Down-regulation of ROBO2 Expression in Prostate Cancers

**DOI:** 10.1007/s12253-013-9722-1

**Published:** 2013-11-24

**Authors:** Youn Jin Choi, Nam Jin Yoo, Sug Hyung Lee

**Affiliations:** Department of Pathology, College of Medicine, The Catholic University of Korea, 505 Banpo-dong, Socho-gu, Seoul, 137-701 South Korea

**Keywords:** ROBO2, Prostate cancer, Expression

## Abstract

Several lines of evidence exist that axon guidance genes are involved in cancer pathogenesis. Axon guidance genes *ROBO1* and *ROBO2* are candidate tumor suppressor genes (TSG). The aim of our study was to address whether ROBO1 and ROBO2 expressions are altered in prostate cancers (PCA). In this study, we analyzed ROBO1 and ROBO2 expressions in 107 PCAs. In the immunohistochemistry, loss of ROBO2 expression was identified in 66 % of PCAs and was significantly higher than that in normal cells (*p <* 0.001). By contrast, there was no significant difference of ROBO1 expression between normal and PCAs. Our results indicate that axon guidance protein ROBO2 is frequently lost in PCA and that ROBO2 might be involved in PCA pathogenesis as a candidate TSG.

## Introduction

Mounting evidence indicates that signaling pathways implicated in development are altered in tumorigenesis as well [1]. SLIT proteins bind ROBO proteins and are involved in axon guidance during development [2]. In addition, SLIT/ROBO interactions play important roles in many processes, including apoptosis, motility, angiogenesis and invasion of cancer cells [2]. For example, decrease of SLIT/ROBO interaction leads to loss of E-cadherin expression [3, 4]. ROBO1-deficient mice suffer from cancer development [5]. Axon guidance genes ROBO1 and ROBO2 are frequently lost in many cancers (head/neck, breast, lung, kidney and uterine cancers), and considered tumor suppressor genes (TSG) in them [6–9]. In prostate cancers (PCA), mRNA expression of axon guidance genes were frequently altered [10]. ROBO1 mRNA expression was down-regulated compared with normal prostate tissues, while ROBO2 mRNA expression was not altered [10]. However, it remains unclear whether ROBO1 and ROBO2 expression is altered in PCA at protein level. In this study, we analyzed expression of ROBO1 and ROBO2 proteins in PCA tissues.

## Materials and Methods

For this, tissue microarray (TMA) blocks containing normal and PCA tissues of 107 patients were used. The PCA were surgically resected 107 adenocarcinomas, and consisted of one Gleason score 5, 10 score 6, 47 score 7, 10 score 8 and 39 score 9 cancers. In addition, prostate intraepithelial neoplasia (PIN) were included in the TMA of 20 patients’ specimens. Age of the patients ranged 43–77 years with an average of 67.6 years. Sizes of the cancers ranged 1.1–5.0 cm in diameter with an average of 2.5 cm. Approval was obtained from the institutional review board for this study.

Using TMA tissue section series, immunohistochemistry for ROBO1 and ROBO2 were performed using ImmPRESS System (Vector Laboratories, Burlingame, CA, USA). Antibodies for human for ROBO1 (GeneTex, Irvine, CA, USA; dilution 1/400) and ROBO2 (Santa Cruz Biotechnology, Santa Cruz, CA, USA; dilution 1/50) were used as primary antibodies. After deparaffinization, heat-induced epitope retrieval was conducted by immersing the slides in Coplin jars filled with 10 mmol/L citrate buffer (pH 6.0) and boiling the buffer for 30 min in a pressure cooker (Nordic Ware, Minneapolis, MN, USA) inside a microwave oven at 700 W; the jars were then cooled for 20 min. Reaction products were developed with diaminobenzidine and counterstained with hematoxylin. Other procedures were performed as described previously [11–13]. Under light microscope, tumors were interpreted as positive when 20–100 % of the cells showed moderate to intense cytoplasm and/or nuclear staining, and as negative when 0–19 % of the cells showed staining by immunohistochemistry. The results were reviewed independently by two pathologists. As negative controls, a slide was treated by replacement of primary antibody with the blocking reagent. The immunostaining was judged to be specific by absence of consistent immunostaining of cells by replacement of primary antibody with the blocking reagent. Also, reduction of signal intensity was observed as dilution of the antibody was increased. For the statistical analysis of the immunohistochemical data, we used *χ* ^2^ and Fisher’s exact tests.

## Results and Discussion

**Fig. 1:**
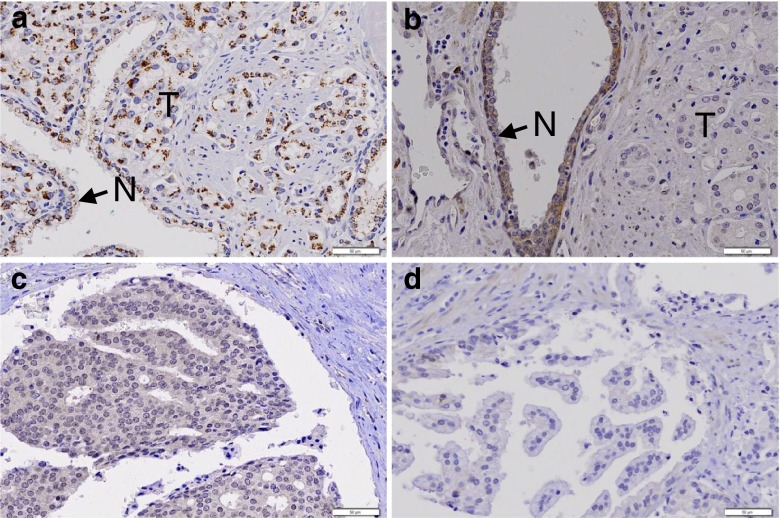
Visualization of ROBO1 and ROBO2 expressions in prostate cancer tissues by immunohistochemistry. **a** Both normal prostate epithelial cells (N) and cancer cells (T) are positive for ROBO1 immunostaining. **b** A prostate cancer shows negative ROBO2 immunostaining in the cancer cells (T), whereas normal epithelial cells (N) are positive for ROBO2 immunostaining. **c** Another prostate cancer shows positive ROBO2 immunostaining in the cancer cells. **d** A PIN lesion shows negative ROBO2 immunostaining in the cells

In the PCA, immunopositivity for ROBO1 was observed in 102 (95 %) of the 107 PCAs (Fig. 1a). Normal prostate glandular cells displayed positive ROBO1 immunostaining in all cases (Fig. 1a). There was no significant difference of ROBO1 expression between normal and PCA (Fisher’s exact test, *p >* 0.05). PIN lesions showed ROBO1 expression in all cases. By contrast, ROBO2 expression was positive only in 36 of the PCA (34 %), while normal prostate glandular cells displayed positive ROBO1 immunostaining in all cases (Fig. 1b and c). ROBO2 expression in PCA was significantly higher than that in normal cells (Fisher’s exact test, *p <* 0.001). PIN showed ROBO2 expression in 40 % of the cases (Fig. 1d). ROBO2 expression was significantly different between normal and PIN (Fisher’s exact test, *p <* 0.001), but not different between PCA and PIN (Fisher’s exact test, *p >* 0.05). Next, we analyzed relationship between ROBO2 expression and pathologic parameters (age, tumor size, vascular invasion, Gleason score and stage). However, we were not able to find any significant association (*χ* ^2^ test, *p* > 0.05).

Because a previous study [2] showed loss of ROBO1 protein in many cancer types and an earlier study [10] showed loss of ROBO1 mRNA expressed compared with normal prostate, we expected to find loss of ROBO1 in PCA. However, we found no difference of ROBO1 protein expression between normal and PCA tissues. Unexpectedly, however, we found that ROBO2 expression is lost in 2/3 of the PCA. Our study also identified that PIN were negative for ROBO2 expression, suggesting that decrease of ROBO2 expression might occur at an early stage of PCA development. In addition to expressional alterations, somatic mutations of both *ROBO1* and *ROBO2* have been reported in pancreatic cancers and fluke-associated cholangiocarcinomas [14, 15]. Recent whole-exome sequencing analyses identified neither *ROBO1* or *ROBO2* mutation in PCA [16, 17], suggesting that somatic mutation of *ROBO1* and *ROBO2* genes may be rare in PCA. In conclusion, our data suggest that loss of axon guidance molecule ROBO2, rather than ROBO1, might be involved in PCA tumorigenesis as a TSG.
